# Secondary Open Aortic Procedure Following Thoracic Endovascular Aortic Repair: Meta‐Analytic State of the Art

**DOI:** 10.1161/JAHA.117.006618

**Published:** 2017-09-13

**Authors:** Ivancarmine Gambardella, George A. Antoniou, Francesco Torella, Cristiano Spadaccio, Aung Y. Oo, Mario Gaudino, Francesco Nappi, Matthew A. Shaw, Leonard N. Girardi

**Affiliations:** ^1^ Department of Cardiothoracic Surgery Liverpool Heart and Chest Hospital Liverpool United Kingdom; ^2^ Information Department Liverpool Heart and Chest Hospital Liverpool United Kingdom; ^3^ Department of Vascular and Endovascular Surgery The Royal Oldham Hospital Manchester United Kingdom; ^4^ Vascular and Endovascular Service Royal Liverpool University Hospital Liverpool United Kingdom; ^5^ Department of Cardiothoracic Surgery Golden Jubilee National Hospital Glasgow United Kingdom; ^6^ Department of Cardiothoracic Surgery Weill Cornell Medicine New York NY; ^7^ Cardiac Surgery Center Cardiologique du Nord de Saint‐Denis Paris France

**Keywords:** aorta, aortic arch, aortic disease, aortic dissection, aortic surgery, Cardiovascular Surgery

## Abstract

**Background:**

Thoracic endovascular aortic repair is characterized by a substantial need for reintervention. Secondary open aortic procedure becomes necessary when further endoluminal options are exhausted. This synopsis and quantitative analysis of available evidence aims to overcome the limitations of institutional cohort reports on secondary open aortic procedure.

**Methods and Results:**

Electronic databases were searched from 1994 to the present date with a prospectively registered protocol. Pooled quantification of pre/intraoperative variables, and proportional meta‐analysis with random effect model of early and midterm outcomes were performed. Subgroup analysis was conducted for patients who had early mortality. Fifteen studies were elected for final analysis, encompassing 330 patients. The following values are expressed as “pooled mean, 95% confidence interval.” Type B dissection was the most common pathology at index thoracic endovascular aortic repair (51.2%, 44.4–57.9). The most frequent indication for secondary open aortic procedure was endoleak (39.7%, 34.6–45.1). More than half of patients had surgery on the descending aorta (51.2%, 45.8–56.6), and one fourth on the arch (25.2%, 20.8–30.1). Operative mortality was 10.6% (7.4–14.9). Neurological morbidity was substantial between stroke (5.1%, 2.8–9.1) and paraplegia (8.3%, 5.2–13.1). At 2‐year follow‐up, mortality (20.4%, 11.5–33.5) and aortic adverse event (aortic death 7.7%, 4.3–13.3, tertiary aortic open procedure 7.4%, 4.0–13.2) were not negligible.

**Conclusions:**

In the secondary open aortic procedure population, type B dissection was both the most common pathology and the one associated with the lowest early mortality, whereas aortic infection and extra‐anatomical bypass were associated with the most ominous prognosis.

**Clinical Perspective**

**What Is New?**

- This meta‐analysis aimed to offer the best evidence possible regarding secondary open aortic procedure (SOAP) after thoracic endovascular aortic aneurysm repair up to date.
- The majority of SOAP is represented by patients initially treated with thoracic endovascular aortic aneurysm repair for chronic type B aortic dissection.
- SOAP for retrograde type A aortic dissection has substantially better outcomes than primary open repair of anterograde type A dissection.
- In presence of infection, SOAP has an ominous prognosis.

**What Are the Clinical Implications?**

- This meta‐analysis corroborates the warning, as expressed by lower‐rank evidence from institutional series in the literature, against the liberal use of thoracic endovascular aortic aneurysm repair to treat chronic type B aortic dissection.
- This meta‐analysis corroborates the warning, as expressed by lower‐rank evidence from institutional series in the literature, against the liberal deployment of an endovascular stent into an infected field.
- SOAP often involves an extensive tract of the thoraco‐abdominal aorta, with complex organ protection strategies: the necessity to guarantee the substantial human and structural resources required for these procedures demands referral to and treatment in tertiary and quaternary aortic centers of excellence.

## Introduction

Pathology of the aortic arch and descending thoraco‐abdominal aorta has historically been one of the most challenging entities in cardiovascular surgery. Conventional open repair of the aortic arch^1^ and the descending thoracic aorta^2^ were pioneered more than 60 years ago. Indications of open repair are well established by now,^3^, ^4^ and its outcomes have constantly improved over time with optimization of organ protection.^5^, ^6^ The dawn of thoracic endovascular aortic repair (TEVAR) was 4 decades later.^7^ Over time, endovascular repair extended its application to aortic pathology other than aneurysm (eg, dissection), and the acronym TEVAR has been adopted to conventionally indicate an endoluminal treatment of the thoracic aorta. Indications for TEVAR have been debated and molded over the past 2 decades, and its outcomes have been shown to be related to compliance with guidelines.^3^, ^4^

One of the main drawbacks of TEVAR compared with open repair is an increased rate of secondary aortic procedures, which is reported between 11% and 15% in recent literature.^8^, ^9^ In some instances, the secondary intervention can be pursued again with an endoluminal approach (secondary endovascular aortic procedure), but when this is not feasible, conversion to open repair (secondary open aortic procedure, SOAP) is required.

SOAP differs from the primary open repair in terms of the following:

*Indications*—SOAP is indicated either to address native aortic pathology not resolved by TEVAR (eg, aneurysm expansion and progression), or new pathological entity as a complication of TEVAR (eg, stent migration/collapse, endoleak), or a pathological entity whose incidence is increased by TEVAR (eg, retrograde type A aortic dissection—[RTAD], esophageal fistula).

*Aortic segments*—SOAP encompasses either aortic segments that have been inadequately treated by TEVAR (eg, descending thoracic aorta replacement for aneurysm expansion), or segments subsequently involved in the pathologic process that TEVAR cannot address (eg, thoraco‐abdominal aorta replacement for aneurysm progression), or segments that need to be replaced as a consequence of TEVAR (eg, aortic arch replacement for type IA endoleak).

*Operative conduct*—SOAP has to be conducted taking into account anatomo‐pathologic specimens altered by the previous TEVAR (eg, suturing a compound native aorta‐stent), circulatory support modifications as a consequence of previous TEVAR (eg, organ perfusion strategies and circulatory arrest consequent to extension of repair behind the native aortic pathology), or concomitant procedures to address extraluminal complications of previous TEVAR (eg, esophageal or pulmonary resection for stent erosion).

As opposed to primary open repair, SOAP has scant reports in the literature. This is because SOAP is a relatively new procedure created by the advent of TEVAR, its incidence is lower compared with primary open repair, and centers with the necessary human expertise and structural resources necessary to perform it are few.

Our aim was to systematically review all the available information on SOAP in the literature, and to overcome the intrinsic limitations of individual series with relatively small populations. This was obtained with a quantitative analysis of the gathered data, with pooling of pre/peri/postoperative data of SOAP and meta‐analysis of its immediate and medium‐term outcomes.

### Objectives

The questions being addressed in the systematic review were framed according to the PICOS (Participants, Interventions, Comparisons, Outcomes, Study design) component of Preferred Reporting Items for Systematic Reviews and Meta‐Analyses:

*Participants*—Patients (no age/sex limitation) undergoing SOAP after TEVAR because:

1. TEVAR failed to treat index pathology, which eventually required SOAP.
2. TEVAR causes complication necessitating SOAP.
3. Further TEVAR cannot address pathologic process in aortic tract contiguous to stented segment.

*Interventions*—SOAP performed on:

1. Vascular segment previously treated with TEVAR.
2. Vascular segment adjacent to aorta previously treated with TEVAR.

*Comparisons*—not applicable

*Outcomes*—The outcomes of interest were:

1 Primary:
  a Perioperative (in hospital or 30‐day postoperative) mortality, stroke, and paraplegia.
  b Two‐year “all cause” mortality and aortic mortality.
2 Secondary
  a Perioperative (in hospital or 30‐day postoperative) cardiac/renal/respiratory failure and re‐exploration for bleeding.
  b Two‐year tertiary open aortic procedure.

*Study design*—Only original articles were considered for inclusion. No randomized controlled trials were expected, because the comparison component of PICOS is absent. To avoid selective treatment bias, and to obtain a sample population as close as possible to the general population, the following cohort studies were excluded:

1. Series limited to a specific SOAP.
2. Series limited to a specific TEVAR population.
3. Series limited to a specific indication for SOAP.

## Methods

### Study Protocol and Eligibility Criteria

#### Study protocol

Methodology and objectives of this analysis were specified in a study protocol, which was registered with the number 42016047593 at the International Prospective Register of Systematic Reviews in Health and Social Care (PROSPERO), developed and maintained by the Centre for Reviews and Dissemination of the University of York, United Kingdom.^10^ The systematic literature review was undertaken according to the Preferred Reporting Items for Systematic Reviews and Meta‐Analyses guidelines.^11^ The review objectives were to investigate preoperative characteristics, intraoperative strategic conduct, and perioperative (early) and postoperative (late) outcomes of patients undergoing SOAP after failed TEVAR.

#### Eligibility criteria

The criteria followed were as follows:

1. Inclusion criteria: our analysis included original articles describing pre/intra/peri/postoperative variables of patients undergoing SOAP. There was no date limit on publications, and no age or sex limit on patients.
2. Clinical exclusion criteria were the following: series reporting mixed data where the variables regarding patients undergoing SOAP and secondary endovascular aortic procedure could not be distinguished, particularly with no specific and distinguishable indications for and outcomes of SOAP; series not detailing quantitatively the specified inclusion criteria; series limited to a specific subpopulation and so not contributing to derive a comprehensive “full picture” analysis of the whole SOAP population (ie, series focusing only on a specific indication for SOAP; series focusing only on a specific SOAP; series encompassing only a specific TEVAR population).
3. Nonclinical exclusion criteria were as follows: overlapping series (only the latest publication on serial reports of a certain cohort was included); no original article (ie, review, case report, editorial).

### Information Sources, Search Strategy, Study Selection, Data Collection Process, and Data Items

#### Information source

Multiple electronic health databases (Medline, Embase, Ovid, Cochrane Library, Google Scholar) were searched from 1994 (advent of TEVAR) to the present date during the review period (September 21, 2016 to December 31, 2016).

#### Search strategy

The databases were searched with an unrestricted search strategy, applying exploded Medical Subject Headings (MeSH) and keywords combined with the Boolean operators AND or OR to retrieve relevant reports: “failed TEVAR” (MeSH); “thoracic endovascular repair”; “TEVAR”; “open reintervention”; “open conversion” (MeSH); “endovascular aneurysm repair”; “open aortic surgery” (MeSH); “TEVAR complication”; “retrograde type A dissection”; “endoleak”; “aneurysm progression”; “aortic stent infection”; “aortic stent migration”; “aortic stent collapse”; “TEVAR secondary intervention.” A second‐level search included a manual screen of the reference lists of the articles identified through the electronic search.

#### Study selection

Eligibility assessment was performed independently in an unblinded standardized manner by 2 reviewers (I.G. and C.S.); disagreements between reviewers were resolved by majority consensus with a third reviewer (M.G.).

#### Data collection process

Data retrieved from the primary sources were entered into a spreadsheet, which was pilot‐tested in 3 randomly selected articles and refined accordingly. One author extracted the data from the included studies (I.G.) and a second author checked the extracted information (C.S.). Disagreements were resolved by discussion with a third author (M.G.). Data were identified in published material only.

Some authors did not report follow‐up data in a fashion suited for our statistical analysis purposes, as stated in the “O” component of PICOS and specified in the prospectively registered protocol. In some instances the information was present but sparse in a narrative form throughout the text (describing morbidity and mortality of individual patients over time, sometimes in a fragmented fashion between the sections “Results” and “Discussion”), rather than extrapolated and explicitly stated in an alphanumeric fashion (eg, in a table): in those cases we retrieved the necessary information by methodically reading the text narrative about each patient reported and so deriving the outcome of interest at 2‐year follow‐up. In other instances, when even text narrative was not helpful, we contacted the relevant institution and asked the following questions: (1) What is the number of “all‐cause death” events at 2‐year follow‐up in the surgical survivors (ie, operative mortality excluded)? (2) What is the number of “aortic deaths” events at 2‐year follow‐up in the surgical survivors (ie, operative mortality excluded)? (3) What is the number of “tertiary aortic operations” events at 2‐year follow‐up? (4) What is the number of patients lost to follow‐up? If there were patients lost to follow‐up, we asked to try to recontact them and fill out the missing data. When this was not achievable, we excluded those data.

#### Data items (with definitions and abbreviations)

The extracted information was divided into 7 categories:

1. Series details: first author; year of publication; journal; study period; number of SOAP.
2. Preoperative variables: age; male sex; elapsed time TEVAR‐SOAP; nonelective cases; comorbidities.
3. Pathology at index TEVAR: degenerative aneurysm; dissection, total; type A dissection, total; acute type A dissection; chronic type A dissection; type B dissection, total; acute type B dissection; chronic type B dissection; coarctation; transection; pseudoaneurysm; perforating aortic ulcer; miscellanea, total (coarctation+transection+pseudoaneurysm+ perforating aortic ulcer); infection (fistula+mycotic aneurysm), total; fistula; mycotic aneurysm.
4. SOAP indication: retrograde type A aortic dissection (RTAD); unstable aneurysm, total (aneurysm expansion+progression+rupture); aneurysm expansion; aneurysm progression, aneurysm rupture; endoleak, total (endoleak I+III); endoleak I; endoleak III; infection, total (infected stent+fistula); infected stent; fistula; miscellanea, total (sum of the following SOAP indications); aortic regurgitation; stent migration; wall perforation; stent collapse; recurrent coarctation; pseudoaneurysm; major branch occlusion; stent thrombosis; stent maldeployment.
5. Intraoperative variables: proximal segment, total; aortic valve; ascending aorta; middle segment, total; proximal hemi‐arch; total arch; total arch+elephant trunk; total arch+frozen elephant trunk; distal hemi‐arch; distal thoracic segment, total; proximal cerclage; distal cerclage; proximal+distal cerclage; descending thoracic segment; thoraco‐abdominal segment; middle+distal thoracic segment, total; distal hemi‐arch+distal thoracic; total arch+descending thoracic; abdominal segment; hybrid, total; proximal debranching; distal debranching; extra‐anatomical; cardiac additional procedure, total; aortic valve; mitral valve; coronary artery bypass grafting; noncardiac additional procedure, total; pulmonary plasty/resection; esophageal plasty/resection; sternotomy, thoracotomy/spiral (thoraco‐phreno‐laparotomy); sternotomy+thoracotomy; sternotomy+laparotomy; clamshell; laparotomy; circulatory support, total; cardiopulmonary bypass no circulatory arrest; cardiopulmonary bypass+circulatory arrest (CA); left heart bypass.
6. Operative variables (within the same admission of or 30 days after SOAP): mortality; multiorgan failure; cardiac failure; respiratory failure; abdominal ischemia; exsanguination; morbidity, total; stroke; cardiac morbidity; renal morbidity; respiratory morbidity; permanent spinal cord injury; recurrent laryngeal nerve paralysis; chylothorax; fistula; aortic infection; nonfatal bleeding. Exsanguination included operative hemorrhage that was fatal either directly, or indirectly consequent to massive blood transfusion leading to fatal end‐organ failure.^12^
7. Adverse events at 2‐year follow‐up: overall (from any cause) mortality; aortic (exclusively related to aortic adverse event) mortality; tertiary open aortic procedure.

### Risk of Bias Assessment

Selective treatment bias was excluded a priori, since series limited to only a specific type of SOAP, a specific type of TEVAR population, or a specific indication for SOAP were not included in the analysis. Methods of assessment of publication bias are described in the Data Synthesis section below.

The methodological quality of observational cohort studies was assessed with the Newcastle‐Ottawa scale.^13^ Using the tool, each study was judged on 8 items, categorized into 3 groups: the selection of the study groups; the comparability of the groups; and the ascertainment of outcome of interest. Stars awarded for each quality item served as a quick visual assessment. Stars were awarded such that the highest quality studies were awarded up to 9 stars. Furthermore, the system developed by the Grades of Recommendation, Assessment, Development and Evaluation (GRADE) working group was used for grading the quality of evidence as high, moderate, low, and very low, based on within‐study risk of bias, directness of evidence, heterogeneity, precision of effects estimates, and risk of population bias.^14^ The risk of bias assessment of the selected studies was performed by 2 authors (I.G. and C.S.).

### Data Synthesis

#### Summary measures

Pooled variables were reported as the mean and 95% confidence interval (CI).

#### Methods of analysis

A preoperative profile and intraoperative treatment conduct of the total SOAP population were obtained by data pooling of category 1 to 5 variables of subpopulations from the included series using simple descriptive statistics. Early and late outcomes of SOAP were obtained by meta‐analyzing data from category 6 to 7 variables of the subpopulations from the included series. The pooled proportion was calculated as the back transformation of the weighted mean of the transformed proportions. We anticipated considerable clinical between‐study heterogeneity and therefore, we applied the random effects model proposed by DerSimonian and Laird.^15^ The unit of analysis was the individual patient.

#### Assessment of heterogeneity

Interstudy heterogeneity was initially assessed visually using the forest plots. Furthermore, we examined heterogeneity with the combination of the Cochran's Q (χ^2^) test and the I^2^ statistic. *P*<0.05 was considered significant for heterogeneity.^16^ Moreover, we considered I^2^ values <50% as indicative of low heterogeneity, I^2^ values between 50% and 75% as indicative of moderate heterogeneity, and I^2^ values >75% as indicative of significant heterogeneity.

#### Assessment of reporting bias

We constructed funnel plots and evaluated their symmetry to visually assess publication bias, as long as a sufficient number of studies (>10) was available. Furthermore, we calculated the Egger's regression intercept to formally assess reporting bias in our review.^17^

#### Sensitivity analysis

Heterogeneity and robustness of pooled proportions were explored by conducting sensitivity analyses. Specifically, we undertook sensitivity analyses to assess the contribution of risk of bias.

#### Subgroup analysis

Variables from the 4 categories “pathology at index TEVAR,” “indication for SOAP,” “aortic segment & procedure,” and “cause of death” were specifically extrapolated for each patient who had early death after SOAP.

“Done to Dead” ratios (DDR) for each of the 4 categories were calculated as follows: denominator=number of SOAPs performed on all patients with a specific variable/numerator=number of patients with the same variable who had early death post‐SOAP.

#### Statistical software

We used the Comprehensive Meta‐Analysis (CMA) software (Biostat, Englewood, NJ).

## Results

### Study Selection

A total of 201 studies were identified according to the search strategy specified above. This initial pool was screened for clinical exclusion criteria, and the following number of articles was accordingly eliminated: 143 for absence of SOAP; 25 for selective indication for SOAP; 2 for selective TEVAR population; 1 for no distinction SOAP/secondary endovascular aortic procedure; 1 for no SOAP outcomes.

**Figure 1:**
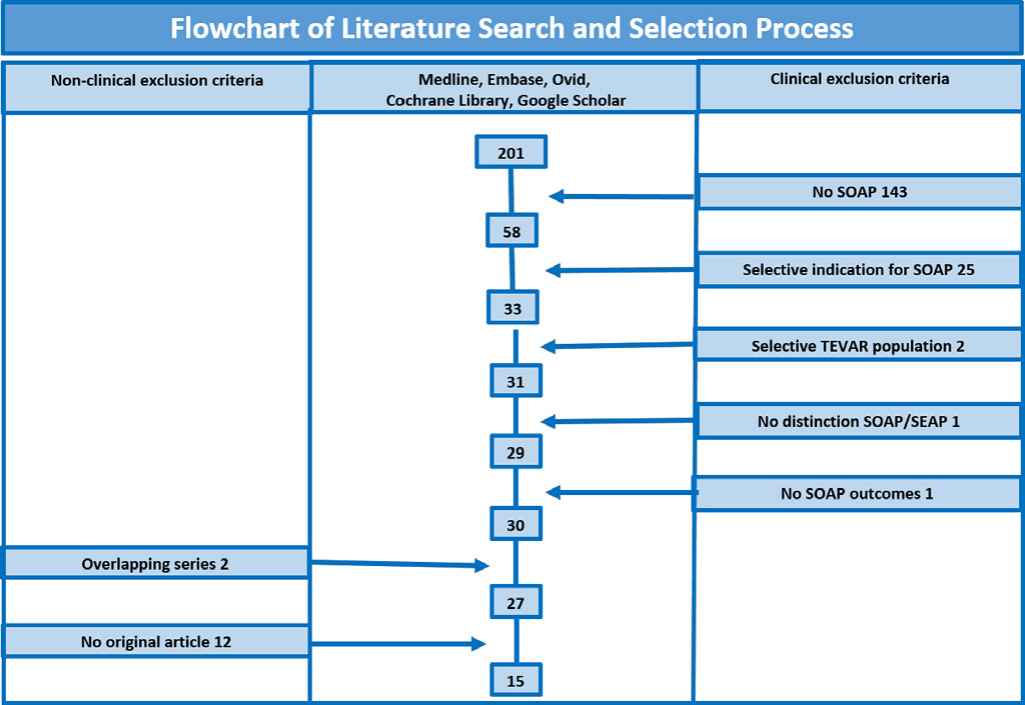
Flowchart of literature search and selection process. SEAP indicates secondary endovascular aortic procedure; SOAP, secondary open aortic procedure; TEVAR, thoracic endovascular aortic repair.

The remaining pool of 29 articles was screened for nonclinical exclusion criteria, and the following number of articles was accordingly eliminated: 2 for overlapping series, 12 for no original article. A flowchart of the literature search and selection process is available in Figure 1. Finally, the 15 articles listed in Table 1 were deemed suitable to be included in our qualitative and quantitative analysis.^8^, ^9^, ^18^, ^19^, ^20^, ^21^, ^22^, ^23^, ^24^, ^25^, ^26^, ^27^, ^28^, ^29^, ^30^

**Table 1:** Details of the Series Included in the Final Synopsis and Meta‐Analysis

Series Included in Synopsis and Meta‐Analysis							
Author	Year of Publication	Journal	Study Period	SOAP, n	Institutions Involved	Secondary Intervention Investigated	TEVAR Source
Canaud^18^	2013	EJVES	2002–2012	12*	Mono‐center	SOAP	Internal
Dumfarth^19^	2011	ATS	1996–2010	21	Bi‐center	SOAP	Internal
Ehrlich^20^	2008	JTCVS	1996–2004	16	Multi‐center	SOAP	Internal
Geisbüsch^21^	2011	JVS	1997–2010	25†	Mono‐center	SOAP+SEAP	Internal
Girdauskas^22^	2008	JTCVS	2002–2007	14	Mono‐center	SOAP	Internal
Grabenwoger^23^	2004	EJCTS	1996–2003	4	Mono‐center	SOAP	Internal
Langer^24^	2008	JVS	2001–2007	8	Bi‐center	SOAP	Internal & External
LeMaire^25^	2012	ATS	1996–2011	35	Mono‐center	SOAP	Internal & External
Mellissano^26^	2016	JCVS	1999–2015	30	Mono‐center	SOAP	Internal
Miyahara^27^	2014	ATS	2000–2012	16	Mono‐center	SOAP+SEAP	Internal & External
Nozdrzykowski^8^	2016	EJCTS	2002–2013	25	Mono‐center	SOAP+SEAP	Internal
Roselli^28^	2014	ATS	2001–2012	50	Mono‐center	SOAP	Internal & External
Scali^29^	2014	JVS	2004–2011	48†	Mono‐center	SOAP+SEAP	Internal
Szeto^9^	2013	JTCVS	2000–2012	20	Mono‐center	SOAP+SEAP	Internal
Zipfel^30^	2007	ATS	1999–2005	6‡	Mono‐center	SOAP+SEAP	Internal & External
Total	2004–2016	···	1996–2015	330	···	···	···

ATS indicates Annals of Thoracic Surgery; EJCTS, European Journal of Cardio‐thoracic Surgery; EJVES, European Journal of Vascular and Endovascular Surgery; JCVS, Journal of Cardiovascular Surgery; JTCVS, Journal of Thoracic and Cardiovascular Surgery; JVS, Journal of Vascular Surgery; SEAP, secondary endovascular aortic procedure; SOAP, secondary open aortic procedure; TEVAR, thoracic endovascular aortic repair.

Specifications: *two patients were excluded for absence of actual SOAP (aortobronchial fistulas treated primarily with TEVAR, wrapping of the stent, and pulmonary resection); ^†^the categories “open” and “hybrid” repair post TEVAR in these series were grouped together as SOAP; ^‡^one patient was excluded for absence of stent deployment before open procedure.

### Study Characteristics

**Figure 2:**
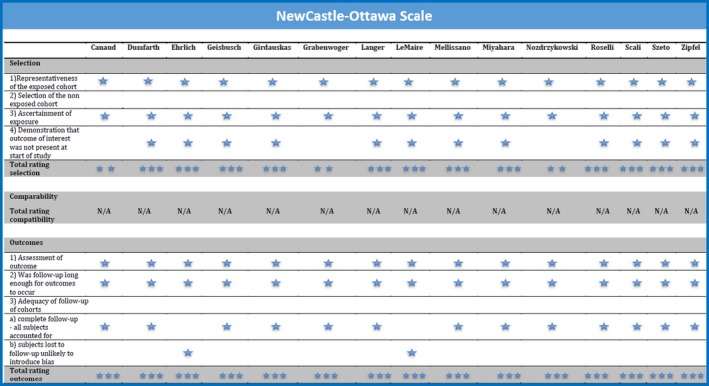
Methodological quality assessment of the series selected for qualitative and quantitative analysis with Newcastle‐Ottawa scoring system.

All of the 15 articles finally selected for qualitative and quantitative synthesis were retrospective studies. The series were published between 2004 and 2016, reporting on SOAP performed between 1996 and 2015. The number of SOAP performed in each series ranged from 4 to 50, and the total number of SOAP reported by all of the series as a whole was 330. Because of absence of SOAP, 2 patients from Canaud's series (aortobrochial fistulas treated primarily with TEVAR, wrapping of the stent, and pulmonary resection), and 1 patient from Zipfel's series (open procedure performed in absence of deployed TEVAR) were excluded. The categories of “open repair” and “hybrid repair” in the series of Geisbusch and Scali were grouped together as SOAP. The reported series were multi‐institutional in 1 article (Ehrlich), bi‐institutional in 2 articles (Dumfarth), and mono‐institutional in the remaining 12 articles. The number of series focusing only on SOAP was 9, whereas 6 series presented mixed but distinguishable variables for SOAP and secondary endovascular aortic procedure. The TEVAR source was internal (SOAP following TEVAR performed at the same institution) in 10 series, and mixed (SOAP following TEVAR performed at the same or other institution) in 5 series. A summary of the studies’ characteristics is detailed in Table 1. The total Newcastle‐Ottawa quality score for each of the included cohort studies reached the highest score possible (range between 5 and 6 stars), considering that the section of comparability was not applicable. The methodological quality of all included studies is depicted in Figure 2.

### Preoperative Variables

#### Clinical characteristics

Adequate data on this variable were available from 11 studies, including 212 patients^18^, ^19^, ^20^, ^22^, ^23^, ^24^, ^25^, ^26^, ^27^, ^28^, ^30^ (Table 2). Patients were on average in their sixth decade of life (62.13, 51.7–77.0 years), and were of male sex in 61.3% of cases (95% CI, 0.541–0.680). This population had a significant comorbidity burden. The vast majority of patients were hypertensive (71.3%, 95% CI, 60.5–80.0), and half of them either were current (37.5%, 95% CI, 28.5–47.5) or past (21.9%, 95% CI, 14.8–31.1) smokers. About one third of the population had significant cardiac (35.4%, 95% CI, 26.6–45.4) and/or respiratory (30.2%, 95% CI, 21.9–40.0) and/or vascular (30.0%, 95% CI, 21.1–40.8) morbidity. SOAP was performed with a mean elapsed time of almost 2 years (20.14, 0.3–61.5 months) from TEVAR, and in a nonelective setting in 34.6% of patients (95% CI, 27.7–42.2). Mean follow‐up was slightly over 2 years (26.08, 10.5–50 months).

**Table 2:** Preoperative Variables of Patients Undergoing SOAP Post TEVAR

Preoperative Variables					
Clinical Characteristics		Pathology at Index TEVAR		SOAP Indication	
Available Patients: 212 Patients		Available Patients: 207 Patients		Available Patients: 330 Patients	
Age, y	62.13 (51.7–77.0)	Degenerative aneurysm	0.343 (0.282–0.410)	Endoleak, total	0.397 (0.346–0.451)
Male sex, n	0.613 (0.541–0.680)	Type A dissection, Total	0.010 (0.003–0.035)	Endoleak I	0.394 (0.334–0.458)
Elapsed time, mo	20.14 (0.3–61.5)	Acute Type A dissection	0.005 (0.001–0.027)	Endoleak III	0.004 (0.001–0.024)
Nonelective, n	0.346 (0.277–0.422)	Chronic Type A dissection	0.005 (0.001–0.027)	Unstable aneurysm	0.212 (0.171–0.259)
Diabetes mellitus	0.100 (0.052–0.185)	Type B dissection, total	0.512 (0.444–0.579)	Aneurysm expansion	0.029 (0.015–0.055)
Hypertension	0.713 (0.605–0.800)	Acute Type B dissection	0.166 (0.116–0.232)	Aneurysm progression	0.150 (0.113–0.197)
Cardiac	0.354 (0.266–0.454)	Chronic Type B dissection	0.312 (0.245–0.388)	Aneurysm rupture	0.021 (0.010–0.046)
Cerebrovascular	0.113 (0.060–0.200)	Infection, total	0.039 (0.020–0.074)	Infection, total	0.179 (0.141–0.224)
Respiratory	0.302 (0.219–0.400)	Fistula	0.024 (0.010–0.055)	Infected stent	0.077 (0.052–0.112)
Renal	0.163 (0.098–0.258)	Mycotic aneurysm	0.005 (0.001–0.027)	Fistula	0.090 (0.063–0.128)
Peripheral arterial	0.300 (0.211–0.408)	Miscellanea, total	0.092 (0.060–0.139)	RTAD	0.161 (0.125–0.204)
Smoking	0.375 (0.285–0.475)	Coarctation	0.005 (0.001–0.027)	Miscellanea	0.046 (0.028–0.074)
Ex‐smoking	0.219 (0.148–0.311)	Transection	0.044 (0.023–0.081)	Aortic regurgitation	0.006 (0.002–0.022)
		Pseudoaneurysm	0.019 (0.008–0.049)	Migration	0.003 (0.001–0.017)
		Perforation aortic ulcer	0.024 (0.010–0.055)	Perforation, wall	0.003 (0.001–0.017)
				Collapse, stent	0.015 (0.007–0.035)
				Recurrent coarctation	0.003 (0.001–0.017)
				Pseudoaneurysm	0.009 (0.003–0.026)
				Major branch occlusion	0.000 (0.000–0.012)
				Stent thrombosis	0.003 (0.001–0.017)
				Stent maldeployment	0.003 (0.001–0.017)

Specifications: Elapsed time, time elapsed between TEVAR and SOAP; unstable aneurysm, sum of the 3 variables below it; infection, total, sum of the 2 variables below it; miscellanea, sum of the remaining variables below it. All variables are expressed as “pooled estimates (95% confidence interval)”, except age and elapsed time, which are expressed as “overall mean (means’ range).” RTAD indicates retrograde type A aortic dissection; SOAP, secondary open aortic procedure; TEVAR, thoracic endovascular aortic repair.

#### Pathology at index TEVAR

**Figure 3:**
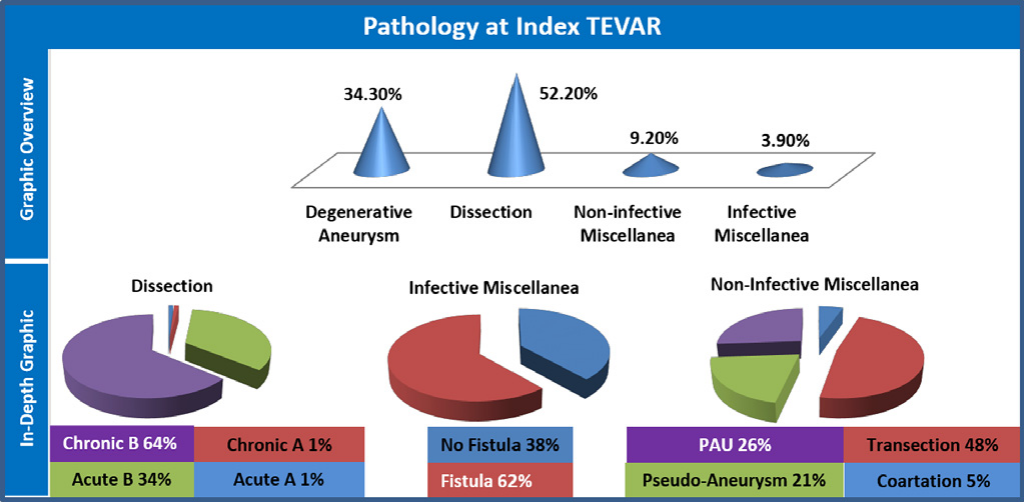
Graphic overview: the upper half of the figure depicts a cone histogram distribution of the pathologic entities at index TEVAR, in patients who subsequently underwent SOAP. In‐depth graphic: the lower half of the figure depicts pie chart proportions of the subsets composing the pathologic entities of dissection, infective, and noninfective miscellanea. Miscellanea indicates every pathologic entity that is not aneurysm or dissection. PAU, Penetrating Aortic Ulcer; SOAP, secondary open aortic procedure; TEVAR, thoracic endovascular aortic repair.

Adequate data on this variable were available from 11 studies, including 207 patients.^8^, ^18^, ^19^, ^20^, ^22^, ^23^, ^24^, ^25^, ^27^, ^28^, ^30^ These data are expressed alphanumerically in Table 2 and graphically in Figure 3. Dissection was the most common pathology at index TEVAR, in patients who subsequently required SOAP. Whereas the contribution of type A dissection was negligible (1.0%, 95% CI, 0.3–3.5), type B aortic dissection was the original pathology in more than half of patients who subsequently needed TEVAR (51.2%, 95% CI, 44.4–57.9). In the series providing specifications about the subgroup of type B dissection, chronic (31.2%, 95% CI, 24.5–38.8) was more problematic compared with acute dissection (16.6%, 95% CI, 11.6–23.2). Degenerative aneurysm was the second most common aortic pathology at the time of TEVAR: it affected about one third of patients who subsequently needed SOAP (34.3%, 95% CI, 28.2–41.0). A stent was deployed in a known infected field in a small but not negligible portion of TEVARs (3.95, 95% CI, 2.0–7.4), sometimes in the presence of a fistulous tract to the aerodigestive tract (2.4%, 95% CI, 1.0–5.5). A miscellanea of other pathologies participated in a minority of cases (9.2%, 95% CI, 6.0–13.9), the most frequent being transection in this group (4.4%, 95% CI, 2.3–8.1).

#### Indications for SOAP

**Figure 4:**
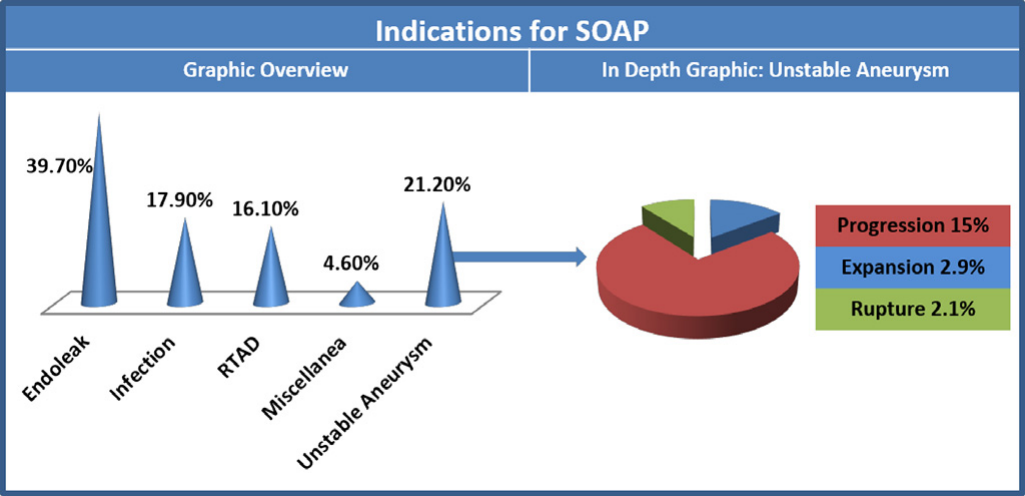
Graphic overview: the left half of the figure depicts a cone histogram distribution of the indications for SOAP. In depth graphic: the right half of the figure represents pie chart proportions of the subsets composing the category “unstable aneurysm.” Expansion indicates further aneurysmal degeneration of segment previously stented; progression, aneurysmal degeneration of segments adjacent to previously stented segment; RTAD, retrograde type A dissection; rupture, rupture of previously stented segment; SOAP, secondary open aortic procedure.

Adequate data on this variable were available from all of the 15 studies, including 330 patients.^8^, ^9^, ^18^, ^19^, ^20^, ^21^, ^22^, ^23^, ^24^, ^25^, ^26^, ^27^, ^28^, ^29^, ^30^ These data are expressed alphanumerically in Table 2 and graphically in Figure 4. The most common indication for SOAP was endoleak (39.7%, 95% CI, 34.6–45.1), which were all type IA with the exception of a single type III. The second most common indication was unstable aneurysm (21.2%, 95% CI, 17.1–25.9), with the main contribution given by aneurysmal progression (15.0%, 95% CI, 11.3–19.7) rather than expansion or rupture. The third most common indication was infection (17.9%, 95% CI, 14.1–22.4), which was complicated by a fistula in about half of cases (9.0%, 95% CI, 6.3–12.8). The fourth most common indication was RTAD (16.1%, 95% CI, 12.5–20.4). A miscellanea of other indications accounted for a small portion of patients (4.6%, 95% CI, 2.8–7.4).

### Intraoperative Variables

**Figure 5:**
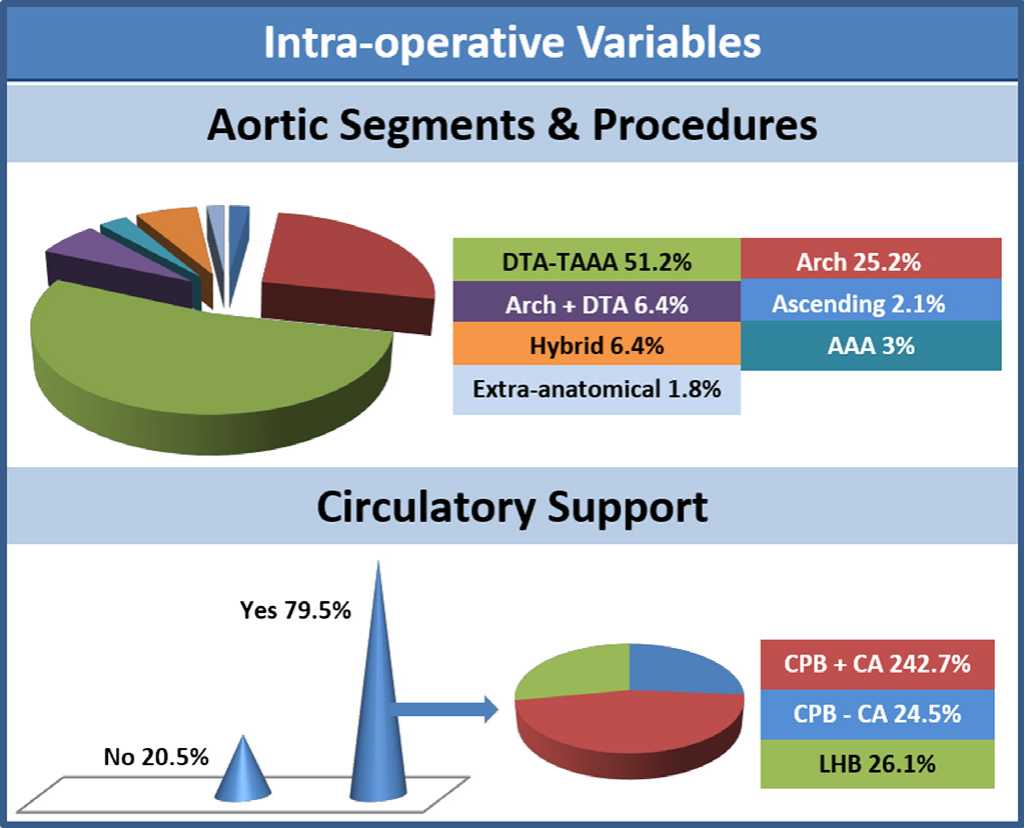
The upper half of the figure depicts a pie graph distribution of the aortic segments and procedures in patients undergoing SOAP. The lower half of the figure provides a cone histogram distribution of the circulatory support required on the left, and pie graph proportions of the types of such circulatory support on the right. AAA indicates abdominal aortic aneurysm; CA, circulatory arrest; CPB, cardiopulmonary bypass; DTA, descending thoracic aneurysm; LHB, left heart bypass; SOAP, secondary open aortic procedure; TAAA, thoraco‐abdominal aortic aneurysm.

Adequate data on this variable were available from all of the 15 studies, including 330 patients.^8^, ^9^, ^18^, ^19^, ^20^, ^21^, ^22^, ^23^, ^24^, ^25^, ^26^, ^27^, ^28^, ^29^, ^30^ These data are expressed alphanumerically in Table 3; a graphic representation of the distributions of aortic procedures and circulatory support is provided in Figure 5.

**Table 3:** Intraoperative Variables of Patients Undergoing Secondary Open Aortic Procedure Post Thoracic Endovascular Repair

Intraoperative Variables			
Available Patients: 330		Available Patients: 330	
Proximal thoracic segment, total	0.021 (0.010–0.043)	Hybrid, total	0.064 (0.042–0.095)
Aortic valve	0.006 (0.002–0.022)	Proximal debranching	0.007 (0.002–0.024)
Root	0.006 (0.002–0.022)	Distal debranching	0.016 (0.007–0.038)
Ascending	0.009 (0.003–0.026)	Extra‐anatomical bypass	0.018 (0.008–0.039)
Middle thoracic segment, total	0.252 (0.208–0.301)	Cardiac concomitant procedure, total	0.000 (0.000–0.016)
Proximal hemiarch	0.046 (0.028–0.074)	Aortic valve	0.036 (0.021–0.063)
Total arch	0.173 (0.136–0.217)	Mitral valve	0.000 (0.000–0.016)
Total arch+elephant trunk	0.009 (0.003–0.026)	Coronary bypass grafting	0.030 (0.017–0.055)
Total arch+frozen elephant trunk	0.003 (0.001–0.017)	Noncardiac concomitant, total	0.046 (0.028–0.076)
Distal hemiarch	0.021 (0.010–0.043)	Pulmonary plasty/resection	0.020 (0.009–0.042)
Distal thoracic (±abdominal) segment, total	0.512 (0.458–0.566)	Esophageal plasty/resection	0.026 (0.013–0.051)
Proximal cerclage	0.006 (0.002–0.022)	Approach	
Distal cerclage	0.018 (0.008–0.039)	Sternotomy	0.282 (0.235–0.335)
Proximal+distal cerclage	0.006 (0.002–0.022)	Thoracotomy/spiral	0.666 (0.611–0.716)
Descending thoracic	0.203 (0.162–0.253)	Sternotomy+thoracotomy	0.012 (0.004–0.034)
Thoraco‐abdominal	0.270 (0.223–0.323)	Sternotomy+laparotomy	0.004 (0.001–0.022)
Middle+distal thoracic segment, total	0.064 (0.042–0.095)	Clamshell	0.004 (0.001–0.022)
Distal hemiarch+descending thoracic	0.052 (0.032–0.081)	Laparotomy	0.033 (0.018–0.059)
Total arch+descending thoracic	0.012 (0.005–0.031)	Circulatory support, total	0.795 (0.723–0.851)
Abdominal segment	0.030 (0.017–0.055)	CPB no circulatory arrest	0.245 (0.183–0.319)
		CPB+circulatory arrest	0.427 (0.365–0.492)
		Left heart bypass	0.261 (0.198–0.336)

The extra‐anatomical bypasses were composed of the following: 1 distal arch to abdominal aorta bypass, 1 ascending to abdominal aorta bypass, 1 middle arch to descending thoracic bypass, 2 arch and visceral debranchings followed by stent extirpation, 1 ascending aorta to left carotid bypass. Specifications and acronyms: CPB indicates cardiopulmonary bypass; spiral, thoraco‐phreno‐laparotomy. All variables are expressed as “pooled estimates (95% confidence interval).”

#### Aortic segments and procedures

More than half of patients were operated on the distal thoracic or thoraco‐abdominal segment (51.2%, 95% CI, 45.8–56.6). About one fourth of patients were operated on the middle thoracic segment (25.2%, 95% CI, 20.8–30.1). The third most common open approach was a more extensive operation involving the middle and distal thoracic segments (6.4%, 95% CI, 4.2–9.5). The hybrid approach was equally frequent, with the number of distal debranching (1.6%, 95% 0.7–3.8) being double the one of proximal debranching (0.7%, 95% CI, 0.2–2.4). The fourth most common segment treated by an open approach was the abdominal aorta (3.0%, 95% CI, 1.7–5.5), followed by the ascending aorta (2.1%, 95% CI, 1.0–4.3). Extra‐anatomical bypass was rare (1.8%, 95% CI, 0.8–3.9), and it was composed of 1 distal arch to abdominal aorta bypass, 1 ascending to abdominal aorta bypass, 1 middle arch to descending thoracic bypass, 2 arch and visceral debranchings followed by stent extirpation, and 1 ascending aorta to left carotid bypass. Cardiac additional procedures were almost equally divided between coronary artery bypass grafting (3.0%, 95% CI, 1.7%–5.5) and aortic valve surgery (3.6%, 95% CI, 2.1%–6.3). The frequency of noncardiac additional procedures was not negligible (4.6%, 95% CI, 2.8%–7.6), because of the necessity of addressing fistulas with plasty/resection of esophagus or bronchi at an almost equal rate.

#### Incision

Two thirds of the population required thoracotomy or thoraco‐phreno‐laparotomy (66.6%, 95% CI, 61.1–71.6), whereas sternotomy was the second most common incision (28.2%, 95% CI, 23.5–33.5). Of the other types of incisions accounting for the minority of the remaining patients, laparotomy was the most common (3.3%, 95% CI, 1.8–5.9).

#### Circulatory support

Total or partial circulatory support was required in the vast majority of cases (79.5%, 95% CI, 72.3–85.1). Circulatory arrest was deemed necessary during full cardiopulmonary bypass in a substantial amount of cases (42.7%, 95% CI, 36.5–49.2%). Partial circulatory support was required in the form of left heart bypass in more than one fourth of cases (26.1%, 95% CI, 19.8–33.6).

### Operative Outcomes

#### Mortality

**Figure 6:**
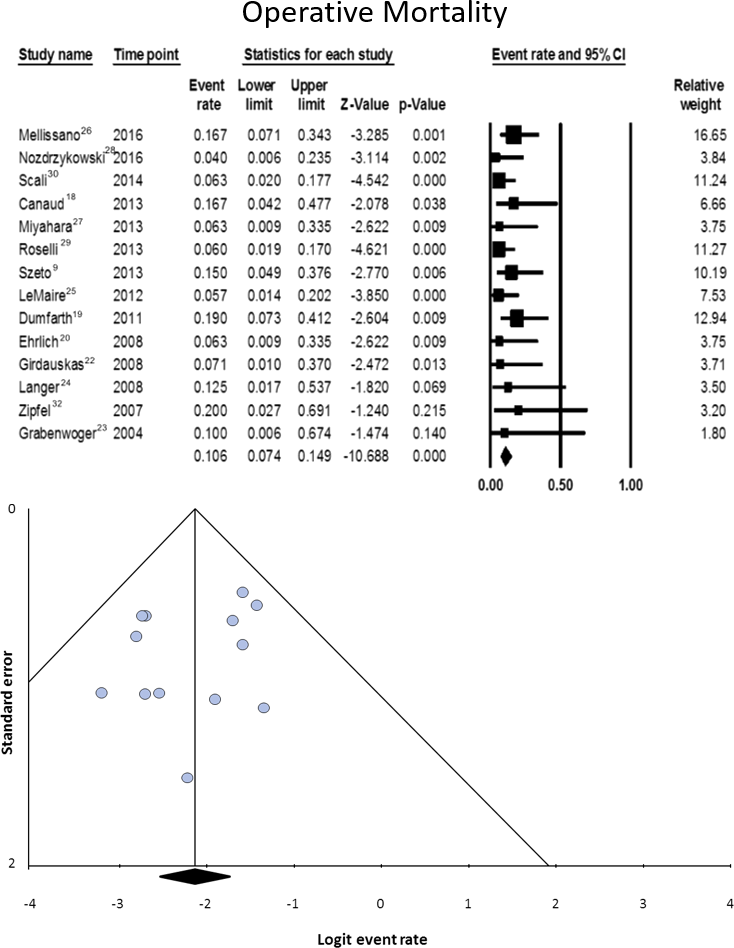
Operative mortality (in hospital or 30 days) in patients undergoing secondary open aortic procedure after thoracic endovascular aortic repair. Data on this variable were available for 14 studies, including 305 patients.^8^, ^9^, ^18^, ^19^, ^20^, ^22^, ^23^, ^24^, ^25^, ^26^, ^27^, ^28^, ^29^, ^30^ The upper half of the figure represents the forest plot of the meta‐analyzed data. The lower half of the figure represents the funnel plot for the assessment of publication bias. CI indicates confidence interval.

Data on this variable were available for 14 studies, including 305 patients.^8^, ^9^, ^18^, ^19^, ^20^, ^22^, ^23^, ^24^, ^25^, ^26^, ^27^, ^28^, ^29^, ^30^ Proportional meta‐analysis showed a pooled rate of 10.6% (95% CI, 7.4–14.9). The pooled estimate likely reflects the true rate, since heterogeneity (I^2^=0%, *P*=0.795) and publication bias (*P*=0.298) were nonsignificant (Figure 6). The most common cause of death was multiorgan failure (7.3%, 95% CI, 3.9–13.0), followed by exsanguination (5.8%, 95% CI, 2.9–11.4) (Table 4).

**Table 4:** Meta‐Analysis of Early (in‐Hospital or 30‐Day) and 2‐Year Adverse Events in Patients Undergoing Secondary Open Aortic Procedure After Thoracic Endovascular Aortic Repair

Meta‐Analysis of Adverse Events	
Operative mortality	
Available patients: 305	
Operative death	0.106 (0.074–0.149)
Cause of death	
Multi‐organ failure	0.073 (0.039–0.130)
Exsanguination	0.058 (0.029–0.114)
Cardiac failure	0.038 (0.016–0.084)
Mesenteric ischemia	0.038 (0.016–0.089)
Operative morbidity	
Available patients: 268	
Stroke	0.051 (0.028–0.091)
Respiratory	0.190 (0.126–0.276)
Cardiac	0.057 (0.029–0.111)
Renal	0.158 (0.117–0.211)
Paraplegia	0.083 (0.052–0.131)
RLN paralysis	0.053 (0.021–0.131)
Chylothorax	0.031 (0.014–0.065)
Fistula	0.031 (0.014–0.066)
Aortic infection	0.031 (0.014–0.066)
Exploration for bleeding	0.050 (0.022–0.112)
Two‐year outcomes	
All‐cause death (174 patients)	0.204 (0.115–0.335)
Aortic death (158 patients)	0.077 (0.043–0.134)
TOAP (144 patients)	0.074 (0.040–0.132)

Variables are expressed as “pooled estimates (95% confidence interval).” RLN, Recurrent Laryngeal Nerve; TOAP, tertiary open aortic procedure.

#### Stroke

**Figure 7:**
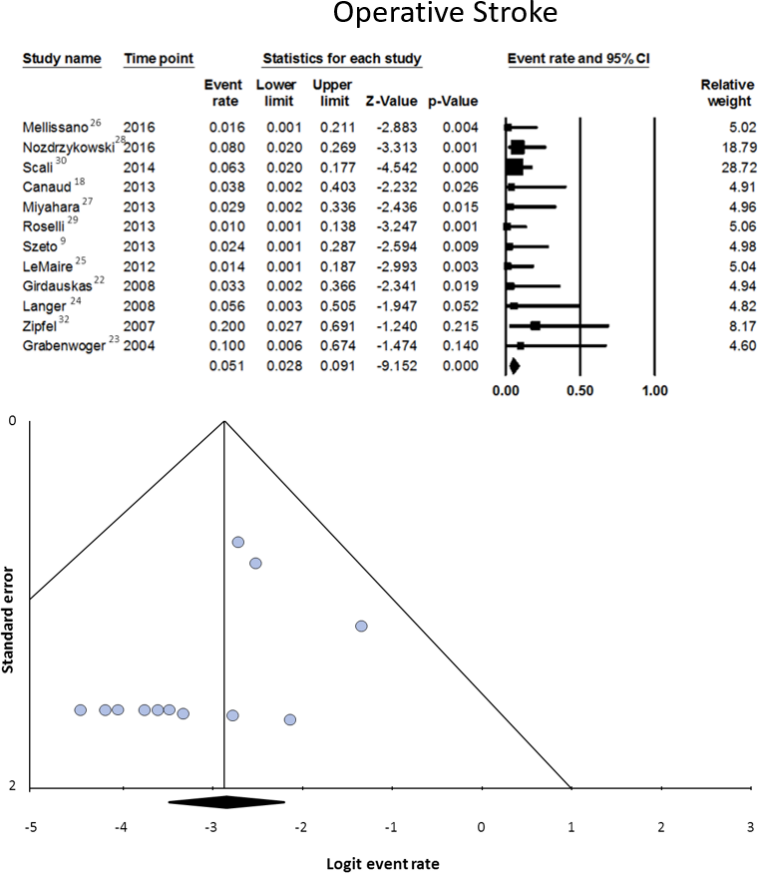
Operative stroke (in hospital or in 30 days) in patients undergoing secondary open aortic procedure after thoracic endovascular aortic repair. Data on this variable were available from 12 studies, involving 268 patients.^8^, ^9^, ^18^, ^22^, ^23^, ^24^, ^25^, ^26^, ^27^, ^28^, ^29^, ^30^ The upper half of the figure represents the forest plot of the meta‐analyzed data. The lower half of the figure represents the funnel plot for the assessment of publication bias.

Data on this variable were available from 12 studies, involving 268 patients.^8^, ^9^, ^18^, ^22^, ^23^, ^24^, ^25^, ^26^, ^27^, ^28^, ^29^, ^30^ Proportional meta‐analysis showed a pooled rate of 5.1% (95% CI, 2.8–9.1). The pooled estimate should reflect the true rate, since heterogeneity (I^2^=0%, *P*=0.854) and publication bias (*P*=0.103) were nonsignificant (Figure 7).

#### Paraplegia

**Figure 8:**
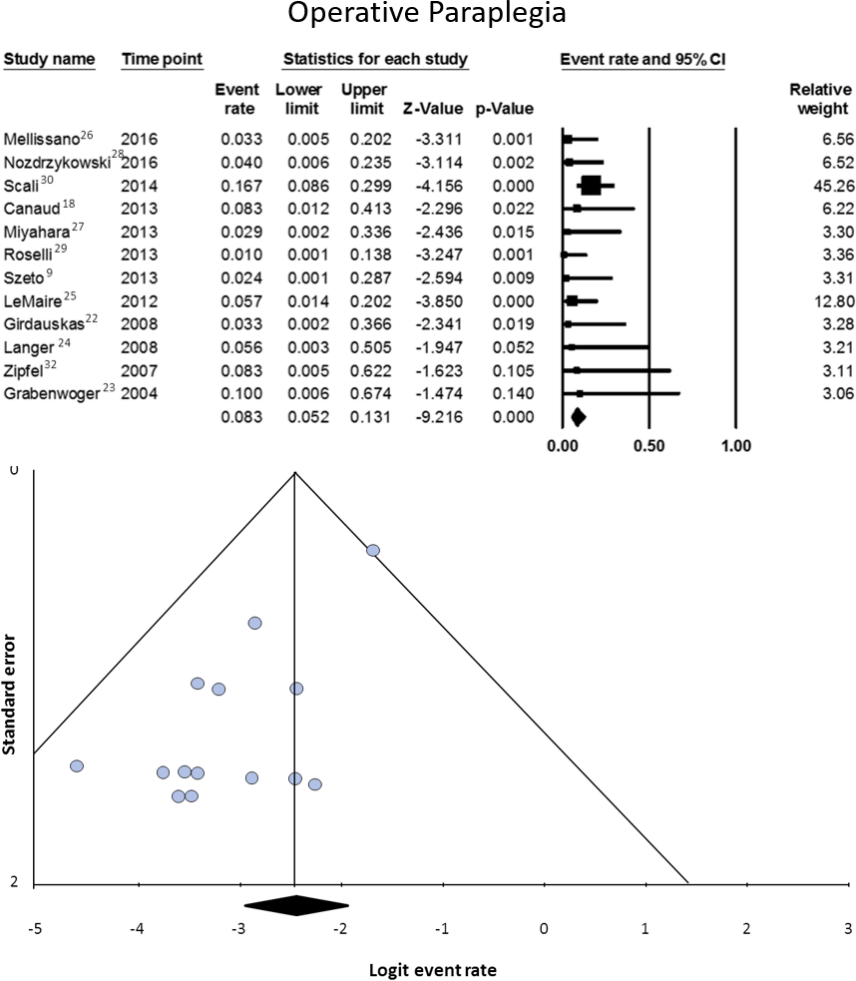
Operative paraplegia (in hospital or in 30 days) in patients undergoing secondary open aortic procedure after thoracic endovascular aortic repair. Data on this variable were available from 12 studies, involving 268 patients.^8^, ^9^, ^18^, ^22^, ^23^, ^24^, ^25^, ^26^, ^27^, ^28^, ^29^, ^30^ The upper half of the figure represents the forest plot of the meta‐analyzed data. The lower half of the figure represents the funnel plot for the assessment of publication bias.

Data on this variable were available from 12 studies, involving 268 patients.^8^, ^9^, ^18^, ^22^, ^23^, ^24^, ^25^, ^26^, ^27^, ^28^, ^29^, ^30^ Proportional meta‐analysis showed a pooled rate of 8.3% (95% CI, 5.2–13.1). Heterogeneity was nonsignificant (I^2^=0%, *P*=0.497), whereas publication bias was significant (*P*=0.001) (Figure 8).

#### End‐organ morbidity

Data on this variable were available from 12 studies, involving 268 patients.^8^, ^9^, ^18^, ^22^, ^23^, ^24^, ^25^, ^26^, ^27^, ^28^, ^29^, ^30^ Proportional meta‐analysis showed a pooled rate for cardiac morbidity of 5.7% (95% CI, 2.9–11.1%) (heterogeneity: *P*=0.228, I^2^=22%; publication bias: *P*=0.001), for respiratory morbidity of 19.0% (95% CI, 12.6–27.6) (heterogeneity: *P*=0.049, I^2^=44%; publication bias: *P*=0.006), and for renal morbidity of 15.8% (95% CI, 11.7–21.1) (heterogeneity: *P*=0.516, I^2^=0%; publication bias: *P*=0.005) (Table 4).

#### Exploration for bleeding

Data on this outcome were available from 12 studies, involving 283 patients.^18^, ^19^ Proportional meta‐analysis showed a pooled rate of 5% (95% CI, 2.2–11.2) (heterogeneity: *P*=0.662, I^2^=0%; publication bias: *P*=0.019) (Table 4).

### Outcomes at 2‐Year Follow‐Up

#### Overall mortality

**Figure 9:**
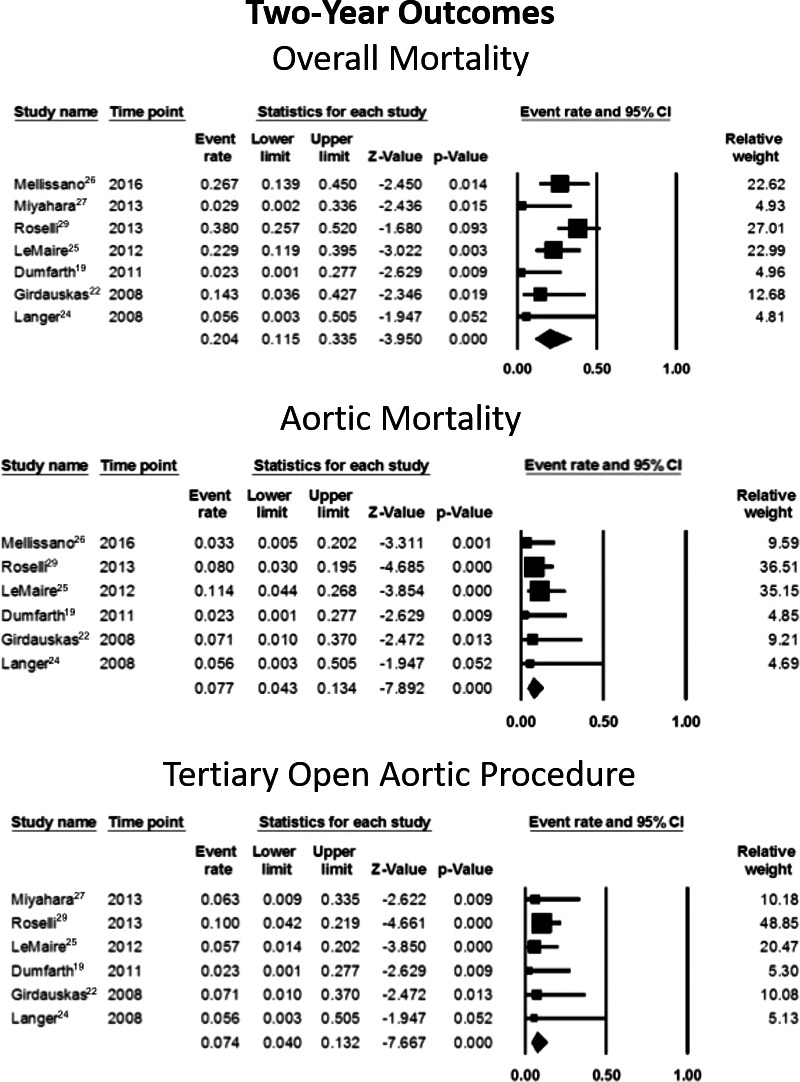
Adverse outcomes at 2‐year follow‐up of secondary open aortic procedure after thoracic endovascular aneurysm repair. Data on overall mortality were available from 7 studies involving 174 patients,^2^, ^5^, ^7^, ^8^, ^9^, ^10^, ^12^ and their meta‐analytic results are depicted in the top forrest plot. Data on aortic mortality were available from 6 studies involving 158 patients,^2^, ^5^, ^7^, ^8^, ^9^, ^12^ and their meta‐analytic results are depicted in the middle forest plot. Data on tertiary aortic open procedure were available from 6 series involving 144 patients,^2^, ^5^, ^7^, ^8^, ^10^, ^12^ and its meta‐analytic results are depicted in the bottom forest plot.

Data on this variable were available from 7 studies, involving 174 patients.^2^, ^5^, ^7^, ^8^, ^9^, ^10^, ^12^ Proportional meta‐analysis showed a pooled rate of 20.4% (95% CI, 11.5–33.5) (Figure 9 and Table 4). Heterogeneity was significant (I^2^=53%, *P*=0.047).

#### Aortic mortality

Data on this variable were available from 6 studies, involving 158 patients.^2^, ^5^, ^7^, ^8^, ^9^, ^12^ Proportional meta‐analysis showed a pooled rate of 7.7% (95% CI, 4.3–13.3) (Figure 9 and Table 4). Heterogeneity was nonsignificant (I^2^=0%, *P*=0.807).

#### Tertiary aortic open procedure

Data on this variable were available from 6 series, involving 144 patients.^2^, ^5^, ^7^, ^8^, ^10^, ^12^ Proportional meta‐analysis showed a pooled rate of 7.4% (95% CI, 4.0–13.2) (Figure 9 and Table 4). Heterogeneity was nonsignificant (I^2^=0%, *P*=0.918).

### Sensitivity Analysis

Results of the sensitivity analysis are reported in Table S1. Sensitivity analysis was repeated after excluding studies of lower methodological quality (ie, with a Newcastle‐Ottawa score <6). The latter studies did not provide data regarding some outcomes for the primary analysis, so the pooled effect estimates remained unchanged (Table S1).

### Subanalysis: Patterns in Early Mortality

**Figure 10:**
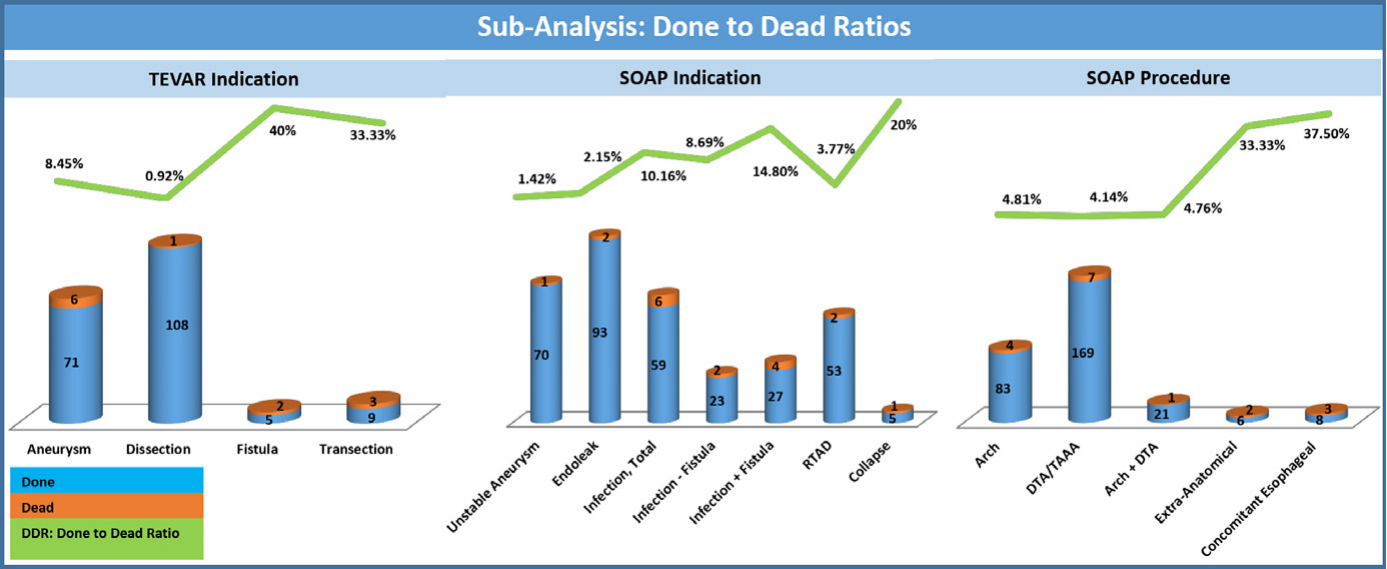
Subanalysis of patients who had early death (ie, in hospital or 30‐day mortality) after secondary open aortic procedure (SOAP) post thoracic endovascular aortic repair (TEVAR). The subanalysis focused on TEVAR indication (left graph), SOAP indication (middle graph), and SOAP procedure (right graph). Color code: the number in the blue portion of the columns expresses the census of patients with a certain variable; the number in the orange portion of the columns expresses the census of the same patients who had early death; the percentage along the green lines expresses the ratio of the 2 previous numbers (done to dead ratio, DDR). DTA indicates descending thoracic aorta; RTAD, retrograde type A dissection; TAAA, thoraco‐abdominal aortic aneurysm.

Adequate data on this variable were available from 8 studies, including 161 patients.^1^, ^2^, ^3^, ^4^, ^5^, ^6^, ^7^, ^8^, ^9^, ^10^, ^11^, ^12^, ^13^, ^14^, ^15^ These data are expressed alphanumerically in Table 5 and graphically in Figure 10.

**Table 5:** Subanalysis of Early Mortalities in Patients Undergone SOAP After TEVAR

Subanalysis of Early Mortalities				
Available Patients: 161				
	Variable	Done, n	Dead, n	DD, %
Pathology at index TEVAR	Aneurysm	71	6	8.45
	Dissection	108	1	0.92
	Fistula	5	2	40
	Transection	9	3	33.33
SOAP indication	Unstable aneurysm	70	1	1.42
	Endoleak	93	2	2.15
	Infection, total	59	6	10.16
	Infected stent	23	2	8.69
	Fistula	27	4	14.8
	RTAD	53	2	3.77
	Migration	1	1	100
	Collapse	5	1	20
Target segment	Middle thoracic	83	4	4.81
	Distal thoracic	169	7	4.14
	Middle+distal thoracic	21	1	4.76
	Extra‐anatomical bypass	6	2	33.33
	Concomitant esophageal	8	3	37.5

Variables are expressed as absolute numbers (done=census of patients with a certain variable who underwent SOAP; dead=census of the patients with the same variable who died during the operative period) and their ratio (DD=done to dead). RTAD indicates retrograde type A aortic dissection; SOAP, secondary open aortic procedure; TEVAR, thoracic endovascular aortic repair.

#### Pathology at index TEVAR

The deadliest pathology at index TEVAR was fistula (DDR 40%), closely followed by transection (DDR 33.33%). SOAP for degenerative aneurysm had a slightly lower mortality than the population average (DDR 8.45%). Finally, chronic type B dissection had a very low mortality when treated with SOAP (DDR 0.94%).

#### SOAP indication

Stent collapse was the deadliest indication for SOAP (DDR 20%). Infection was the second most deadly indication (DDR 10.16%), especially when accompanied by fistula (14.8%). RTAD had a low mortality compared with the average of the whole population (DDR 3.77).

#### Target segment

Extra‐anatomical bypass was the deadliest SOAP performed (DDR 33.33%). There was no significant difference in mortality when SOAP was performed either on the arch (DDR 4/83, 4.81%), the descending thoracic aorta (DDR 7/169, 4.14%), or a combination of both (DDR 1/21, 4.76%). Mortality increased substantially when a concomitant esophageal procedure was added (37.5%). There were no deaths among SOAP performed on either the proximal aortic segment or the abdominal segments.

#### Cause of death

The most common cause of death was multiorgan failure (50%). Exsanguination was the second most common cause of death (35.71%), directly or indirectly when massive transfusion requirements led to fatal end‐organ failure. One case of mesenteric ischemia and 1 case of acute cardiac failure accounted for the remaining deaths.

## Discussion

### Summary of Evidence

#### Data availability and preoperative considerations

The body of evidence regarding SOAP in the literature is represented mainly by institutional series of small–medium size, and our meta‐analysis aimed to overcome some of their intrinsic limitations. Most series report on SOAP performed on patients who underwent TEVAR both at the same and in another institution. Consequently, this body of evidence analyzes the numerator represented by SOAP, but not the denominator represented by the whole population of patients who underwent TEVAR.

The population undergoing SOAP is relatively young (mean age 62.13 years) and there is no sex skewing (49.69% male). Surgeons usually face SOAP within 2 years (20.14 months) from index TEVAR, and in one third of cases (34.6%, 95% CI, 27.7–42.2) perform it in a nonelective setting.

#### Impact of aortic pathology on SOAP

##### Dissection and SOAP

Dissection is not the prevalent pathology at index TEVAR in the literature,^31^ but it becomes the most prevalent in the portion of patients who subsequently require SOAP. Indeed, type B dissection was the underlying original pathology in more than half of patients who underwent SOAP (51.2%, 95% CI, 44.4–57.9), and in this group chronic dissection (31.2%, 95% CI, 24.5–38.8) was 2 times more problematic than acute dissection (16.6%, 95% CI, 11.6–23.2). This finding confirms what has already been observed in the literature: the presence of a thick indurated intimal flap often prevents full stent expansion and so effective exclusion of the false lumen. The latter circumstance is a potential recipe leading to SOAP (eg, type I endoleak with or without aneurysm expansion, perfusion of the false lumen, migration).^32^ One interesting point emerging from our analysis is that type B dissection is at the same time the most numerous and the least deadly SOAP subgroup. Consequently, even if dissection is the most troublesome indication for TEVAR in terms of primary success, secondary open repair can be attained with the lowest mortality among the whole SOAP population. The latter finding echoes the results of primary open repair in the literature, which are far superior when the etiology is chronic dissection rather than degenerative aneurysm. This consideration should firmly discourage liberal stenting of chronic dissections, which could be treated by primary open repair with both a lower mortality compared with degenerative aneurysm, and a low rate of reintervention that is a key feature in these patients (on average a decade younger than the ones with degenerative aneurysm).^6^

One fourth of patients (26%) do not survive open repair of acute type A dissection according to the International Registry of Acute Aortic dissection,^33^ whereas SOAP for RTAD was performed with low mortality (3.77%) in our pooled population. We could speculate that 3 factors may contribute to the substantially lower mortality of RTAD compared with primary type A dissection. First, a pre‐existing stent distal to the ascending aorta could prevent the increase in mortality caused by postoperative cerebral (odds ratio 2.18, 95% CI, 1.45–3.24, *P*<0.001) and visceral (odds ratio 3.24, 95% CI, 1.94–5.35, *P*<0.001) malperfusion.^34^ Second, a proportion of RTADs may develop chronically, eliminating the mortality of type A dissection related to the acute setting. Third, most of these patients are operated on in aortic centers of excellence, likely because the above postulated less acute presentation allows secondary referral because of the complexity of the cases.

##### Infection and SOAP

Aortic infection was an ominous condition regarding SOAP, with an operative mortality of 10.16% in this subgroup. This figure was graver when the infection was so severe as to be associated with a fistulous communication with the aerodigestive tract, and the longer the fistula was present the higher the operative mortality was: 14.8% when the fistula developed at the time of SOAP, and 40% when the fistula was already diagnosed at the time of TEVAR. Accordingly, operative mortality was 37.5% when SOAP included a concomitant esophageal procedure. Consequently, deploying a stent into an infective field should be highly discouraged, and especially in the presence of fistula the only sensible use of TEVAR should be as a bridge to open repair when clinically indicated.

##### Degenerative aneurysm and SOAP

As pathology at index TEVAR, degenerative aneurysm was substantially less frequent (34.3% versus 52.2%) but almost 10 times more deadly (8.45% versus 0.92%) than dissection in patients who underwent SOAP. Regarding indication for SOAP, aneurysm progression was 5 times more frequent than aneurysm expansion (15% versus 2.9%). This means that the most frequent reason why TEVAR fails to effectively treat aneurysmal pathology is not suboptimal exclusion of the aneurysmal sac. It is rather its inadequacy to address a more extensive pathology, which requires further treatment encompassing various aortic segments over time. This reflects the lack of a reliable and reproducible endovascular solution, to treat extensive aortic pathology involving the epiaortic and visceral tracts.^35^, ^36^

#### Operative considerations

##### Aortic segments and procedures

Although most TEVARs are limited to the descending thoracic aorta,^37^ our analysis shows that when they fail the required SOAP remains confined to the same segment only in one fifth of cases (20.3%). Indeed, an extension of the operative field to either the aortic arch proximally or the thoraco‐abdominal aorta distally is necessary in 58.6% of cases (aortic arch 25.2%, thoraco‐abdominal aorta 27%, aortic arch+descending aorta 6.4%) (Table 3). The subgroup of patients undergoing extra‐anatomical bypass had the highest mortality (33.33%). This is likely a reflection of the fact that extra‐anatomical bypass is often the only option left when all the iterations of conventional repair are not pursuable, and this is reflective of the gravity of the situation.

##### Circulatory support

Involvement of such aortic segments explains the frequent necessity of circulatory support, which was required in 79.5% of cases. A complete form of circulatory support with cardiopulmonary bypass was used in two thirds of patients (67.2%), with the adjunct of circulatory arrest in 42.7% of patients. A partial form of circulatory support with left heart bypass was required in one fourth (26.1%) of patients.

#### Early and medium‐term outcomes

SOAP is a high‐risk procedure, as witnessed by its 10.6% early mortality rate. This is likely a reliable figure, since heterogeneity and publication bias were nonsignificant. The price of frequent necessity to extend the operative field bi‐directionally to arch and thoraco‐abdominal segment is paid with a high incidence of permanent neurological injury, which includes involvement of either the brain (5.1%) or the spinal cord (8.3%).

Of the patients who survived the immediate postoperative period, one fifth (20.4%) did not survive past the 2‐year landmark. The high heterogeneity of this figure (I^2^=53%, *P*=0.047) is likely a reflection of variable accuracy in the follow‐up processes, rather than of variability in surgical risk among subpopulations. Indeed, if the latter was true, the heterogeneity of early mortality would have likely been significant as well. Combining pre‐ and postdischarge mortality, one third of (31%) patients did not survive at 2 years post‐SOAP.

**Figure 11:**
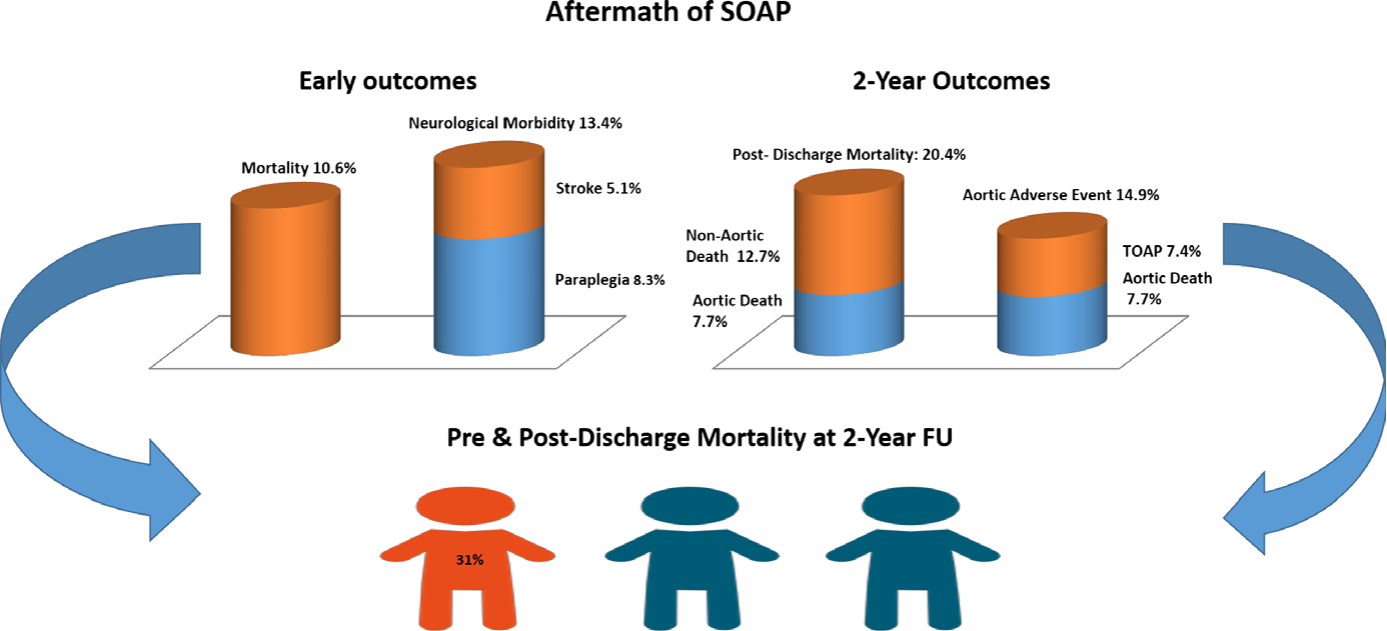
The histograms in the left upper third of the figure represent the rates of early (ie, in hospital or 30‐day) mortality, stroke, and paraplegia after secondary open aortic procedure (SOAP). The histograms in the right upper third of the figure represent the rates of all‐cause death, aortic death, and tertiary open aortic procedure (TOAP) in the survivors of SOAP at 2‐year follow‐up (FU). The lower third of the figure represents the proportion of patients who are dead at 2‐year FU post‐SOAP, summing up pre‐ and postdischarge mortality.

One sixth of patients had further significant aortic complications at 2‐year follow‐up, which led either to tertiary aortic open procedure (7.4%) or aortic death (7.7%) homogeneously across the series (Figure 11).

### Limitations

One intrinsic limitation of our quantitative analysis lies in its material: retrospective cohort studies from institutional databases. Corresponding strengths were a rigorous methodology, ensured by a prospectively registered protocol and Preferred Reporting Items for Systematic Reviews and Meta‐Analyses standards, and a high quality of the retrospective studies included as indicated by Newcastle‐Ottawa assessment. Another limitation could be the potential reporting bias, which for some outcomes is indicated by the statistical analysis. On the other hand, an acknowledged strength is that the majority of included studies report clinically relevant outcomes. Consequently, it should be appropriate to state that this is the best evidence possible to date, since realistically no prospective randomized studies are foreseeable because of the nature of the condition investigated.

## Conclusion

SOAP often extends behind the boundaries of the previously stented aortic segment. Frequent involvement of the arch and thoraco‐abdominal tracts requires complex circulatory support and organ protection strategies, which aim to mitigate the substantial encephalo‐spinal morbidity. Regardless, aortic dissection is the underlying pathology in the majority of SOAPs, and operative mortality is the lowest in this subgroup. Aortic infection and extra‐anatomical bypass carry the most ominous prognosis for SOAP. Multiorgan failure and exsanguination are the main contributors to postoperative mortality. Mortality at 2‐year follow‐up is substantial, driven also by a non‐negligible rate of adverse aortic events (ie, aortic death or tertiary aortic open procedure) in the midterm.

## Disclosures

Mr Torella received research grants from Endologix; is a paid consultant by Endologix; received travel grants from Endologix and Bolton, and received speaker's fees from Abbott. The remaining authors have no disclosures to report.

## Supporting information


**Table S1.** Sensitivity Analysis of the Included Series

## References

[1] DeBakey ME , Crawford ES , Cooley DA , Morris GC Jr . Successful resection of fusiform aneurysm of aortic arch with replacement by homograft. Surg Gynecol Obstet. 1957;105:657–664. 13495827

[2] Lam CR , Aram HH . Resection of the descending thoracic aorta for aneurysm: a report of the use of a homograft in a case and experimental study. Ann Surg. 1951;134:743–752. 14878384 10.1097/00000658-195110000-00019 PMC1802960

[3] Hiratzka LF , Bakris GL , Beckman JA , Bersin RM , Carr VF , Casey DE Jr , Eagle KA , Hermann LK , Isselbacher EM , Kazerooni EA , Kouchoukos NT , Lytle BW , Milewicz DM , Reich DL , Sen S , Shinn JA , Svensson LG , Williams DM . 2010 ACCF/AHA/AATS/ACR/ASA/SCAI/SIR/STS/SVM guidelines for the diagnosis and management of patients with Thoracic Aortic Disease: a report of the American College of Cardiology Foundation/American Heart Association Task Force on Practice Guidelines, American Association for Thoracic Surgery, American College of Radiology, American Stroke Association, Society of Cardiovascular Anesthesiologists, Society for Cardiovascular Angiography and Interventions, Society of Interventional Radiology, Society of Thoracic Surgeons, and Society for Vascular Medicine. Circulation. 2010;121:266–369.

[4] Erbel R , Aboyans V , Boileau C , Bossone E , Bartolomeo RD , Eggebrecht H , Evangelista A , Falk V , Frank H , Gaemperli O , Grabenwöger M , Haverich A , Iung B , Manolis AJ , Meijboom F , Nienaber CA , Roffi M , Rousseau H , Sechtem U , Sirnes PA , Allmen RS , Vrints CJ . 2014 ESC guidelines on the diagnosis and treatment of aortic diseases: document covering acute and chronic aortic diseases of the thoracic and abdominal aorta of the adult. The Task Force for the Diagnosis and Treatment of Aortic Diseases of the European Society of Cardiology (ESC). Eur Heart J. 2014;35:2873–2926. 25173340 10.1093/eurheartj/ehu281

[5] Coselli JS , Green SY . Evolution of aortic arch repair. Tex Heart Inst J. 2009;36:435–437. 19876421 PMC2763466

[6] Coselli JS , LeMaire SA , Preventza O , de la Cruz KI , Cooley DA , Price MD , Stolz AP , Green SY , Arredondo CN , Rosengart TK . Outcomes of 3309 thoracoabdominal aortic aneurysm repairs. J Thorac Cardiovasc Surg. 2016;151:1323–1338. 26898979 10.1016/j.jtcvs.2015.12.050

[7] Dake MD , Miller DC , Semba CP , Mitchell RS , Walker PJ , Liddell RP . Transluminal placement of endovascular stent‐grafts for the treatment of descending thoracic aortic aneurysms. N Engl J Med. 1994;331:1729–1734. 7984192 10.1056/NEJM199412293312601

[8] Nozdrzykowski M , Luehr M , Garbade J , Schmidt A , Leontyev S , Misfeld M , Mohr FW , Etz CD . Outcomes of secondary procedures after primary thoracic endovascular aortic repair. Eur J Cardiothorac Surg. 2016;49:770–777. 26341994 10.1093/ejcts/ezv279

[9] Szeto WY , Desai ND , Moeller P , Moser GW , Woo EY , Fairman RM , Pochettino A , Bavaria JE . Reintervention for endograft failures after thoracic endovascular aortic repair. J Thorac Cardiovasc Surg. 2013;145:165–170. 10.1016/j.jtcvs.2012.11.046 23410774

[10] University of York, Centre for Reviews and Dissemination, York, UK . PROSPERO: international prospective register of systematic reviews. Available at: http://www.crd.york.ac.uk/PROSPERO/. Accessed March 10, 2017.

[11] Liberati A , Altman DG , Tetzlaff J , Mulrow C , Gøtzsche PC , Ioannidis JP , Clarke M , Devereaux PJ , Kleijnen J , Moher D . The PRISMA statement for reporting systematic reviews and meta‐analyses of studies that evaluate healthcare interventions: explanation and elaboration. BMJ. 2009;339:b2700. 19622552 10.1136/bmj.b2700 PMC2714672

[12] Sihler KC , Napolitano LM . Complications of massive transfusion. Chest. 2010;137:209–220. 20051407 10.1378/chest.09-0252

[13] Wells GA , Shea B , O'Connell D , Peterson J , Welch V , Losos M , Tugwell P . The Newcastle‐Ottawa Scale (NOS) for assessing the quality of nonrandomised studies in meta‐analyses. Available at: http://www.ohri.ca/programs/clinical_epidemiology/oxford.asp. Published in 2014. Accessed January 11, 2017.

[14] Guyatt GH , Oxman AD , Vist GE , Kunz R , Falck‐Ytter Y , Alonso‐Coello P , Schünemann HJ ; GRADE Working Group . GRADE: an emerging consensus on rating quality of evidence and strength of recommendations. BMJ. 2008;336:924–926. 18436948 10.1136/bmj.39489.470347.AD PMC2335261

[15] DerSimonian R , Laird N . Meta‐analysis in clinical trials. Control Clin Trials. 1986;7:177–188. 3802833 10.1016/0197-2456(86)90046-2

[16] Higgins JP , Thompson SG , Deeks JJ , Altman DG . Measuring inconsistency in meta‐analyses. BMJ. 2003;327:557–560. 12958120 10.1136/bmj.327.7414.557 PMC192859

[17] Egger M , Davey Smith G , Schneider M , Minder C . Bias in meta‐analysis detected by a simple, graphical test. BMJ. 1997;315:629–634. 9310563 10.1136/bmj.315.7109.629 PMC2127453

[18] Canaud L , Alric P , Gandet T , Ozdemir BA , Albat B , Marty‐Ane C . Open surgical secondary procedures after thoracic endovascular aortic repair. Eur J Vasc Endovasc Surg. 2013;46:667–674. 24138778 10.1016/j.ejvs.2013.08.022

[19] Dumfarth J , Michel M , Schmidli J , Sodeck G , Ehrlich M , Grimm M , Carrel T , Czerny M . Mechanism of failure and outcome of secondary surgical interventions after thoracic endovascular aortic repair (TEVAR). Ann Thorac Surg. 2011;91:1141–1146. 21440134 10.1016/j.athoracsur.2010.12.033

[20] Ehrlich MP , Nienaber CA , Rousseau H , Beregi JP , Piquet P , Schepens M , Bartoli JM , Schillinger M , Fattori R . Short term conversion to open surgery after endovascular stent‐grafting of the thoracic aorta: the Talent thoracic registry. J Thorac Cardiovasc Surg. 2008;135:1322–1326. 18544380 10.1016/j.jtcvs.2007.09.036

[21] Geisbüsch P , Hoffmann S , Kotelis D , Able T , Hyhlik‐Dürr A , Böckler D . Reinterventions during midterm follow‐up after endovascular treatment of thoracic aortic disease. J Vasc Surg. 2011;53:1528–1533. 21609796 10.1016/j.jvs.2011.01.066

[22] Girdauskas E , Falk V , Kuntze T , Borger MA , Schmidt A , Scheinert D , Mohr FW . Secondary surgical procedures after endovascular stent grafting of the thoracic aorta: successful approaches to a challenging clinical problem. J Thorac Cardiovasc Surg. 2008;136:1289–1294. 19026818 10.1016/j.jtcvs.2008.05.053

[23] Grabenwoger M , Fleck T , Ehrlich M , Czerny M , Hutschala D , Schoder M , Lammer J , Wolner E . Secondary surgical interventions after endovascular stent‐grafting of the thoracic aorta. Eur J Cardiothorac Surg. 2004;26:608–613. 15302058 10.1016/j.ejcts.2004.05.003

[24] Langer S , Mommertz G , Koeppel TA , Schurink GW , Autschbach R , Jacobs MJ . Surgical correction of failed thoracic endovascular aortic repair. J Vasc Surg. 2008;47:1195–1202. 18514837 10.1016/j.jvs.2008.01.003

[25] LeMaire SA , Green SY , Kim JH , Sameri A , Parenti JL , Lin PH , Huh J , Coselli JS . Thoracic or thoracoabdominal approaches to endovascular device removal and open aortic repair. Ann Thorac Surg. 2012;93:726–732. 22364967 10.1016/j.athoracsur.2011.10.080

[26] Melissano G , Tshomba Y , Mascia D , Baccellieri D , Kahlberg A , Bertoglio L , Nardelli P , Negri G , Chiesa R . Late open conversion after TEVAR. J Cardiovasc Surg. 2016;57:491–497. 27102628

[27] Miyahara S , Nomura Y , Shirasaka T , Taketoshi H , Yamanaka K , Omura A , Sakamoto T , Inoue T , Minami H , Okada K , Okita Y . Early and midterm outcomes of open surgical correction after thoracic endovascular aortic repair. Ann Thorac Surg. 2013;95:1584–1590. 23566645 10.1016/j.athoracsur.2013.02.027

[28] Roselli EE , Abdel‐Halim M , Johnston DR , Soltesz EG , Greenberg RK , Svensson LG , Sabik JF III . Open aortic repair after prior thoracic endovascular aortic repair. Ann Thorac Surg. 2014;97:750–756. 24411576 10.1016/j.athoracsur.2013.10.033

[29] Scali ST , Beck AW , Butler K , Feezor RJ , Martin TD , Hess PJ , Huber TS , Chang CK . Pathology‐specific secondary aortic interventions after thoracic endovascular aortic repair. J Vasc Surg. 2014;59:599–607. 24571937 10.1016/j.jvs.2013.09.050 PMC4120941

[30] Zipfel B , Hammerschmidt R , Krabatsch T , Buz S , Weng Y , Hetzer R . Stent‐grafting of the thoracic aorta by the cardiothoracic surgeon. Ann Thorac Surg. 2007;83:441–448. 17257967 10.1016/j.athoracsur.2006.09.036

[31] Cao CQ , Bannon PG , Shee R , Yan TD . Thoracic endovascular aortic repair—indications and evidence. Ann Thorac Cardiovasc Surg. 2011;17:1–6. 21587120 10.5761/atcs.ra.10.01612

[32] Thrumurthy SG , Karthikesalingam A , Patterson BO , Holt PJ , Hinchliffe RJ , Loftus IM , Thompson MM . A systematic review of mid‐term outcomes of thoracic endovascular repair (TEVAR) of chronic type B aortic dissection. Eur J Vasc Endovasc Surg. 2011;42:632–647. 21880515 10.1016/j.ejvs.2011.08.009

[33] Hagan PG , Nienaber CA , Isselbacher EM , Bruckman D , Karavite DJ , Russman PL , Evangelista A , Fattori R , Suzuki T , Oh JK , Moore AG , Malouf JF , Pape LA , Gaca C , Sechtem U , Lenferink S , Deutsch HJ , Diedrichs H , Marcos y Robles J , Llovet A , Gilon D , Das SK , Armstrong WF , Deeb GM , Eagle KA . The International Registry of Acute Aortic dissection (IRAD): new insight into an old disease. JAMA. 2000;283:897–903. 10685714 10.1001/jama.283.7.897

[34] Czerny M , Schoenhoff F , Etz C , Englberger L , Khaladj N , Zierer A , Weigang E , Hoffmann I , Blettner M , Carrel TP . The impact of pre‐operative malperfusion on outcome in acute type A aortic dissection: results from the GERAADA registry. J Am Coll Cardiol. 2015;65:2628–2635. 26088302 10.1016/j.jacc.2015.04.030

[35] Ziganshin BA , Elefteriades JA . Surgical management of thoracoabdominal aneurysms. Heart. 2014;100:1577–1582. 25092876 10.1136/heartjnl-2013-305131

[36] Hongku K , Dias NV , Sonesson B , Resch TA . Total aortic endovascular repair. J Cardiovasc Surg. 2016;57:784–805. 27654102

[37] Nation DA , Wang GJ . TEVAR: endovascular repair of the thoracic aorta. Semin Intervent Radiol. 2015;32:265–271. 26327745 10.1055/s-0035-1558824 PMC4540616

