# Supplementary material for: Secondary Open Aortic Procedure Following Thoracic Endovascular Aortic Repair: Meta‐Analytic State of the Art
Source: J Am Heart Assoc. 2017 Sep 13;6(9):e006618. doi: 10.1161/JAHA.117.006618 (PMC5634303; doi:10.1161/JAHA.117.006618)
Supplement: Supplementary file 1 — Table S1. Sensitivity Analysis of the Included Series [file JAH3-6-e006618-s001.pdf]

SUPPLEMENTAL MATERIAL

Table S1. Sensitivity analysis of the included series

	Event rate (95% CI)	Heterogeneity	Publication bias
**Operative outcomes**			
Mortality	0.106 (0.073-0.153)	P=0.707, I^2=0%	P = 0.395
Stroke	0.044 (0.022-0.088)	P=0.713, I^2=0%	-
Paraplegia	0.067 (0.034-0.127)	P=0.284, I^2=18%	-
Cardiac morbidity	0.055 (0.023-0.122)	P=0.127, I^2=36%	-
Respiratory morbidity	0.205 (0.131-0.306)	P=0.043, I^2=50%	-
Renal morbidity	0.159 (0.111-0.223)	P=0.355, I^2=10%	-
Re-exploration for bleeding	0.048 (0.019-0.116)	P=0.473, I^2=0%	-
**Outcomes at 2-year follow up**			
Overall mortality	0.204 (0.115-0.335)	P=0.047, I^2=53%	-
Aortic mortality	0.077 (0.043-0.134)	P=0.807, I^2=0%	-
Tertiary open aortic procedure	0.074 (0.040-0.132)	P=0.918, I^2=0%	-
